# Genetic variations regulate alternative splicing in the 5' untranslated regions of the mouse glioma-associated oncogene 1, Gli1

**DOI:** 10.1186/1471-2199-11-32

**Published:** 2010-04-30

**Authors:** Ramesh Palaniswamy, Stephan Teglund, Matthias Lauth, Peter G Zaphiropoulos, Takashi Shimokawa

**Affiliations:** 1 Department of Biosciences and Nutrition, Karolinska Institutet, Huddinge, SE-14157 Sweden

## Abstract

**Background:**

Alternative splicing is one of the key mechanisms that generate biological diversity. Even though alternative splicing also occurs in the 5' and 3' untranslated regions (UTRs) of mRNAs, the understanding of the significance and the regulation of these variations is rather limited.

**Results:**

We investigated 5' UTR mRNA variants of the mouse Gli1 oncogene, which is the terminal transcriptional effector of the Hedgehog (HH) signaling pathway. In addition to identifying novel transcription start sites, we demonstrated that the expression ratio of the Gli1 splice variants in the 5' UTR is regulated by the genotype of the mouse strain analyzed. The GT allele, which contains the consensus intronic dinucleotides at the 5' splice site of intron 1B, favors exon 1B inclusion, while the GC allele, having a weaker 5' splice site sequence, promotes exon 1B skipping. Moreover, the alternative Gli1 5' UTRs had an impact on translational capacity, with the shorter and the exon 1B-skipped mRNA variants being most effective.

**Conclusions:**

Our findings implicate novel, genome-based mechanisms as regulators of the terminal events in the mouse HH signaling cascade.

## Background

Alternative splicing and transcriptional initiation are key mechanisms, which generate diversity both at the mRNA and protein levels. Recently, several independent research efforts revealed that more than 90% of human genes are alternatively spliced [1,2], and about 50% of both human and mouse genes have multiple alternative promoters [3]. Additionally, a genome-wide screening of alternative splicing and transcriptional initiation estimated that a significant number of genes are differentially spliced within 5' and 3' untranslated regions (UTRs) [4]. Moreover, another genome-wide analysis identified 324 out of 17897 genes that display associations between flanking single nucleotide polymorphisms (SNPs) and gene expression/alternative transcription, demonstrating the regulatory effects of genetic variation in human populations [5]. These non-bias/genome-wide analyses highlight the importance of alternative splicing/promoter usage as general mechanisms of regulation control in mammalian cells.

UTRs are considered to influence gene expression by modulating mRNA stability and/or translational efficiency. Consequently, UTR heterogeneity for a specific gene is likely to have a differential impact on protein expression [6]. Analysis of variable 5' UTRs in the *TGF-beta*, *BRCA1 *and *MDM2 *genes, have indicated that the shorter UTR variants are translated more efficiently [7].

The Hedgehog (HH) signaling pathway plays a central role in embryonic development and adult tissue homeostasis [8]. Abnormal activation of the pathway has been associated with various cancers in skin, brain, lung, digestive tract, prostate and pancreas [9-12]. The mechanistic details of the HH signaling pathway, which is generally thought to be well conserved in evolution, have mostly emerged from studies in Drosophila. In the absence of HH ligands, the PTCH receptor inhibits the activity of the 7-pass transmembrane protein Smoothened (SMO), which acts as a positive regulator of the pathway. Interaction of HH ligands with PTCH relieves the inhibitory control of PTCH on SMO, allowing the GLI transcription factor to dissociate from the negative regulator Suppressor of Fused (SUFU) and translocate into the nucleus, activating target genes. In mammals there are three paralogues of HH proteins, Sonic HH, Indian HH and Desert HH, two PTCH receptors, PTCH1 and PTCH2, and three GLI transcription factors, GLI1, GLI2 and GLI3. Additionally, splice variants of several HH signaling components have been identified [13], in line with an earlier report, which, by the use of genome-wide RNAi, highlighted the importance of alternative splicing in this pathway [14].

GLI1 was originally identified as a highly expressed gene in human glioma [15] and acts as a downstream effector of the HH signaling cascade, mediating the transcriptional response [16]. GLI1 is also a target gene of the HH pathway, resulting in a positive feedback loop. Moreover, overexpression of GLI1 in transgenic mice leads to the induction of basal cell carcinomas and trichoepitheliomas [17]. Alternative splicing in the GLI1 5' UTR regions of human and mouse has earlier been reported by Wang and Rothnagel [18]. Recently, we and others have demonstrated the occurrence of functional, differential splicing events in the coding regions of human GLI1 [19,20].

In this study, novel mouse Gli1 mRNA variants, whose transcriptional initiation is further upstream of the reported exon 1 sequence, were identified. Additionally, we obtained evidence that genetic variation is a key determining factor for the alternative splicing events in the Gli1 5' UTRs, which have functional implications on translational efficiency.

## Results

### Identification of novel transcription start sites in the mouse Gli1 gene

**Figure 1:**
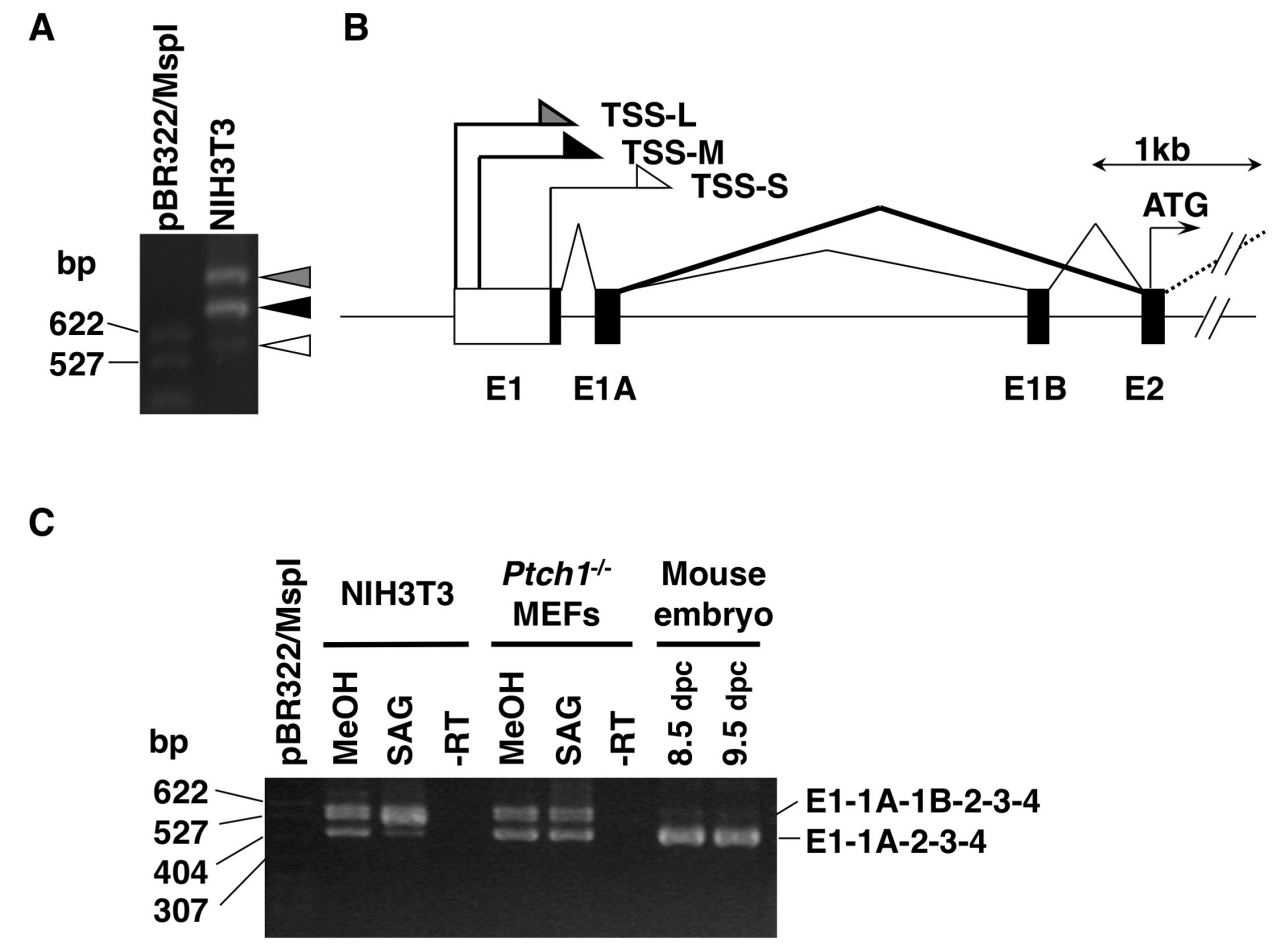
**Alternative transcripts of the mouse Gli1 gene**. (A) Detection of alternative transcription start sites (TSS). RACE analysis was performed on NIH3T3 cells using reverse primers within exon 4 of the Gli1 gene followed by agarose gel electrophoresis. White, black and gray triangles indicate transcripts, which are transcribed from known (TSS-S) or novel (TSS-M and TSS-L) TSSs, respectively. Note that the RACE product indicated by the black triangle also includes a minor variant that is transcribed from TSS-L and skips exon 1B. The MspI digested pBR322 molecular weight markers are also shown. (B) Genomic structure of the mouse *Gli1 *5' UTR region. Exons are represented by black boxes with the splicing pattern indicated. The white box represents the newly identified exon 1 region, which is transcribed from the novel TSSs (black and gray arrows). The exon 1B skipping event is highlighted by a bold line. The initiator methionine codon (ATG) in exon 2 is also shown. (C) Analysis of skipping events of Gli1 5' exons in NIH3T3 cells, *Ptch1*^-/- ^MEFs and mouse embryos. Cells were treated either with methanol (MeOH) or the **S**mo **ag**onist, SAG. Alternative splicing was evaluated by RT-PCR analysis using primer sets within exons 1 and 4. The individual PCR products detected on agarose gel electrophoresis were sequence-verified. The MspI digested pBR322 molecular weight markers are also shown.

In a previous report, we have shown the presence of novel human GLI1 splice variants, which skip exons 2 and 3 [19]. Moreover, Wang and Rothnagel had earlier reported the skipping of exon 1A and/or exon 1B in mouse Gli1 [18]. To clarify the splicing pattern of the mouse Gli1 gene at its 5' end, **R**apid **A**mplificaton of **c**DNA **E**nds (RACE) analysis using reverse primers within exon 4 (Table 1) was performed on RNA extracted from NIH3T3 cells. Surprisingly, we did not detect any RACE product below the expected size of a transcript that includes exon 1, 1A, 1B, 2, 3 and 4 (Figure 1A, white triangle). On the contrary, the major products observed were significantly longer. This finding implies the presence of other exon(s) and/or the extension of known exons. Sequence-verification of the RACE products demonstrated the existence of novel transcriptional start sites (TSSs) for exon 1, which are located approximately 350 (TSS-L) or 290 (TSS-M) nucleotides upstream of the reported Gli1 TSS, TSS-S (Figure 1B, DDBJ: AB234616 and AB234617). Additionally, we analyzed Gli1 exon-skipping events in the HH signaling responsive NIH3T3 cells, with or without treatment by the **S**mo **ag**onist SAG [21], the constitutively activated *Ptch1*^-/- ^mouse embryonic fibroblast (MEF) cell line, and C57BL/6 mouse embryos at 8.5 and 9.5 days post coitum (d.p.c.) by nested PCR with primer sets within exon 1 and 4 (Table 1). Inclusion of all exons (1-1A-1B-2-3-4) and exon 1B skipping were the two splicing events detected in the analyzed samples (Figure 1C). Interestingly, the ratio of these two variants was different depending on context. Moreover, we could neither detect exon 2 and 3 skipping, as observed in human [19], nor skipping of both exons 1A and 1B [18] in these samples or in a mouse cDNA panel (data not shown). Additionally, the recently reported exon 3-partial exon 4 skipping in human [20], was not observed, even though the reverse primers used hybridized to the retained exon 4 segment. Note that all mouse Gli1 mRNA variants observed are characterized by unique 5' untranslated regions (5' UTRs), but a common translational start site in exon 2.

**Table 1:** Primer sequences for RACE and nested PCR analysis

	Forward primers in exon 1	Reverse primers in exon 4
1st PCR	5'-AGTTTCCAGCCCTGGACCAC	5'-GAGGTCCGGATTACGGTTT
Nested PCR	5'-ACCGCGCCCCGACGGAG	5'-ATCAGAAAGGGGCGAGATGG

### Expression profiles of Gli1 variants in embryos, cell lines, and medulloblastoma tumors

**Figure 2:**
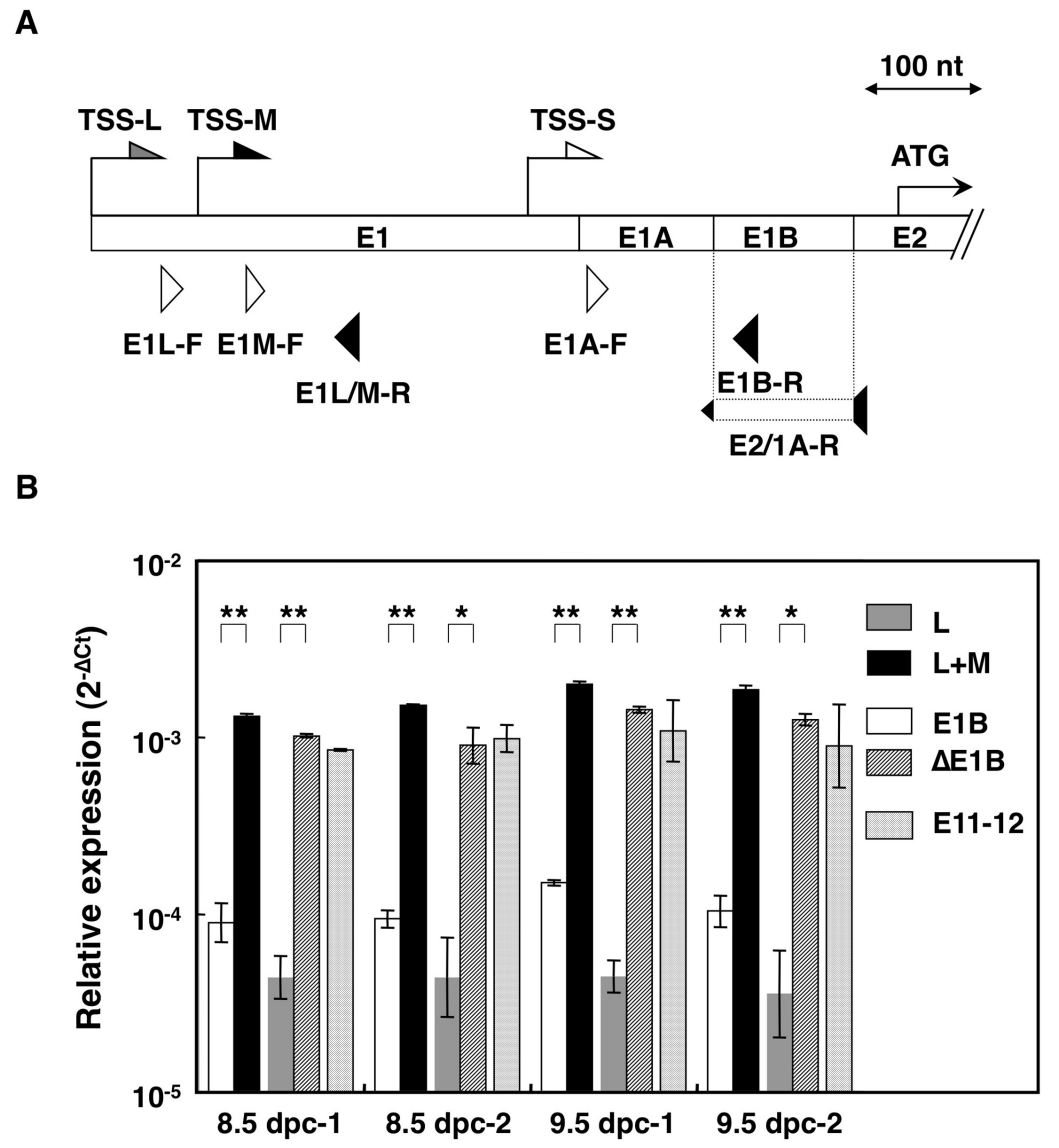
**Expression of Gli1 variants during embryogenesis**. (A) Schematic representation of the variant-specific primer sets for real-time RT-PCR used to detect the alternative Gli1 transcripts. Gray, black and white arrows indicate the alternative transcription start sites TSS-L, TSS-M and TSS-S of the Gli1 mRNAs. The initiator methionine codon (ATG) in exon 2 is also shown. The positions of the forward and reverse primers for detection of the L (E1L-F, E1L/M-R), L+M (E1M-F, E1L/M-R), ΔE1B (E1A-F, E1B-R), and ΔE1B (E1A-F, E2/1A-R) transcripts are indicated by white and black triangles, respectively. Exons are shown by boxes. (B) Gli1 expression profile in mouse embryos. The L, L+M, E1B, and ΔE1B variants were quantified by real-time RT-PCR using SYBR Green in two 8.5 d.p.c. and two 9.5 d.p.c. mouse embryos. Data are presented as relative Ct (**C**ycle **t**hreshold, the number of PCR cycles that reaches an arbitrary threshold) values (ΔCt), that is the Ct of the individual transcripts minus the Ct of the housekeeping gene *Arp*. A logarithmic plot of the 2^-ΔCt ^values is shown. The error bars indicate the standard deviation and the statistical significance between the L and L+M as well as the E1B and ΔE1B transcripts is shown (*: p < 0.05, **: p < 0.01, Student's t-test).

To compare the expression pattern of the 5' Gli1 variants, we performed real-time RT-PCR with PCR primers designed to specifically detect the individual Gli1 transcripts (Figure 2A and Table 2). As a control, we also used another primer set, from exon 11 to exon 12, to detect the 3' end of the Gli1 mRNAs.

**Table 2:** Primer sequences for real-time RT-PCR

	Forward primers	Reverse primers
L	5' **-**CATAAGCCCGGCACCCCCTCTCTA	5' **-**ACCCGCGAGAAGCGCAAACTTTTT
L+M	5' **-**ACGAGGGAAGTGAGCGGGAAGAGC	5' **-**ACCCGCGAGAAGCGCAAACTTTTT
E1B	5' **-**TTGTCCGCGCCTCTCCCACATACTA	5' **-**GGGCAGAAGCAGCCGTTCAGTCTT
ΔE1B	5' **-**TTGTCCGCGCCTCTCCCACATACTA	5'-TCAGGGAAGGATGAGGGGACCTG
E11-12	5' **-**CCCATAGGGTCTCGGGGTCTCAAAC	5'-GGAGGACCTGCGGCTGACTGTGTAA
Arp	5'-TGCACTCTCGCTTTCTGGAGGGTGT	5'-AATGCAGATGGATCAGCCAGGAAGG
Gapdh	5'-GGTGTGAACGGATTTGGCCGTATTG	5'-CCGTTGAATTTGCCGTGAGTGGAGT

Initially, we confirmed Gli1 variant expression in the C57BL/6 mouse embryos at 8.5 and 9.5 d.p.c. by real-time RT-PCR (Figure 2B). Similarly to the nested PCR analysis shown in figure 1B, Gli1 variants skipping exon 1B (ΔE1B) were more abundant to the ones including exon 1B (E1B) in both embryonic stages. Furthermore, the **C**ycle **t**hreshold (Ct) values of Gli1 variants that were transcribed from TSS-L and TSS-M (L+M) were nearly equal to the Ct values of either the ΔE1B or the exon 11 to 12 (E11-12) transcripts. This finding highlighted that the majority of the mouse Gli1 mRNAs initiate at upstream TSSs in the embryo, in agreement with the previous observations of the NIH3T3 cell line (Figure 1B). Additionally, to elucidate the *in vivo *distribution of these Gli1 transcripts in developing embryonic compartments, we analyzed 9.5 d.p.c. mouse embryos by whole mount *in situ *hybridization (See additional file 1). All probes used showed comparable distribution patterns of the variants, as also did a Gli1 3' end control riboprobe. These data suggested that the expression of all the alternative Gli1 mRNAs was likely to be controlled by similar developmental mechanisms.

**Figure 3:**
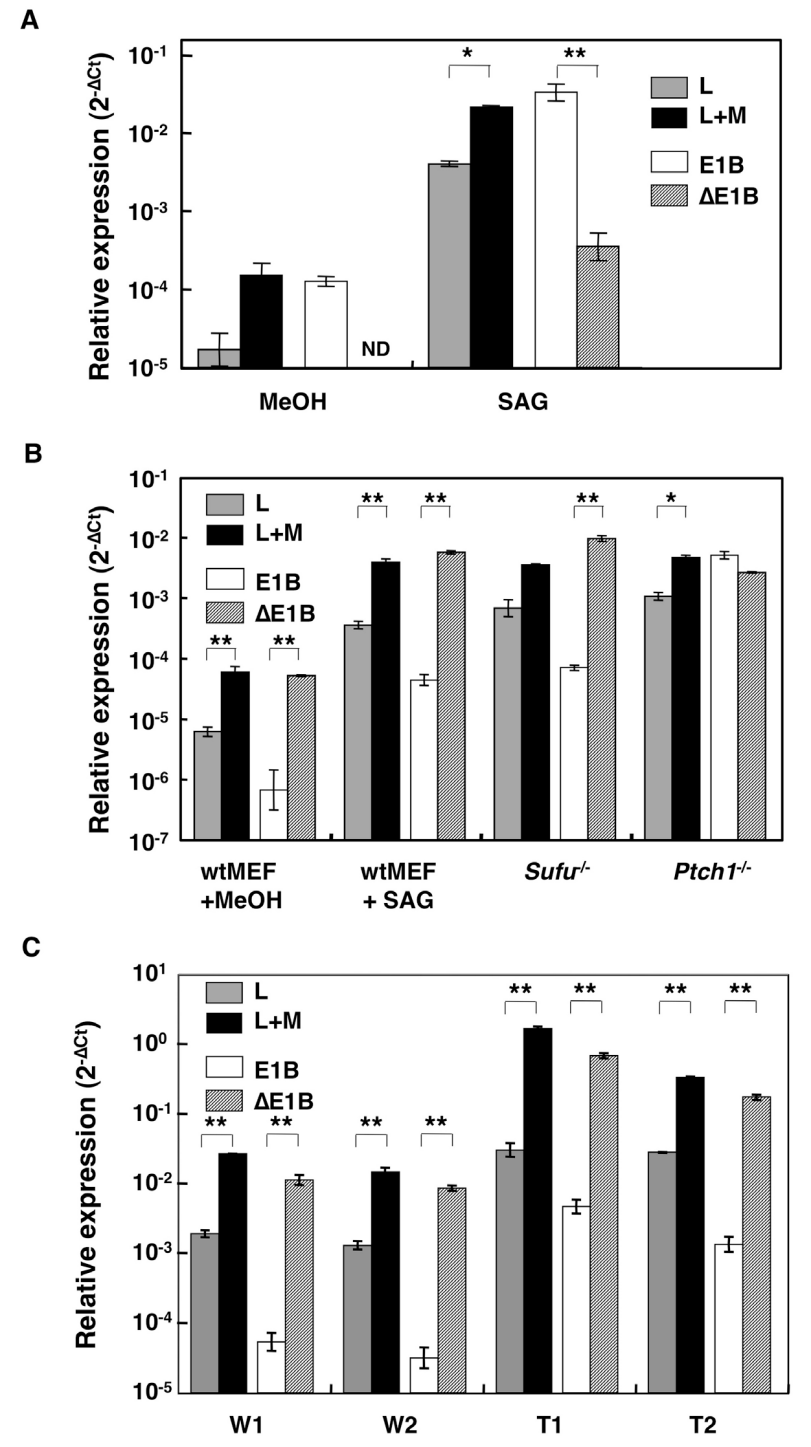
**Upregulation of Gli1 variants by HH signaling activation/tumorigenesis**. Gli1 expression profile in NIH3T3 cells (A), mouse embryonic fibroblasts (MEFs) (B), and medulloblastoma tumors (C). Gli1 variants in NIH3T3 cells or wild type MEFs (wtMEFs), treated with methanol (MeOH) or the **S**mo **ag**onist, SAG, *Ptch1*^-/- ^or *Sufu*^-/- ^MEFs, and normal cerebellum (N1 and N2) or medulloblastoma tumors (MB1 and MB2) from *Sufu*^+/-^*Trp53*^-/- ^mice were analyzed by real-time RT-PCR. Data are presented as relative Ct (**C**ycle **t**hreshold) values (ΔCt), that is the Ct of individual transcripts minus the Ct of the housekeeping genes *Arp */*Gapdh*. A logarithmic plot of the 2^-ΔCt ^values is shown. The PCR primer sets to detect the variants are the ones depicted in Figure 2A. The error bars indicate the standard deviation and the statistical significance between the L and L+M as well as the E1B and ΔE1B transcripts is shown (*: p < 0.05, **: p < 0.01, Student's t-test). ND, non-detected, the signal is below the sensitivity limit of the assay. Note that although real-time RT-PCR can not detect the ΔE1B variant without SAG treatment in NIH3T3 cells, standard nested RT-PCR (Figure 1B) does, but apparently in a non-quantitative pattern.

To test the impact of HH signaling activation on the expression of the Gli1 variants, real-time RT-PCR analysis was performed in the HH signaling responsive NIH3T3 cells and wild type MEFs (wtMEFs), and the constitutively active *Sufu*^-/- ^and *Ptch1*^-/- ^MEFs (Figure 3A and 3B). In NIH3T3 cells, the E1B variant was more abundant than the ΔE1B, and HH signaling activation, elicited by SAG, could not alter the predominance of E1B. On the other hand, wtMEFs expressed the ΔE1B variant as the major Gli1 transcript and, in line with NHI3T3 cells, exhibited a parallel upregulation of the alternative Gli1 variants by HH signaling activation, resulting in an expression pattern similar to that of *Sufu*^-/- ^MEFs. Moreover, *Ptch1*^-/- ^MEFs equally expressed both the E1B and the ΔE1B variants, even though the Ct value of the L+M transcripts was similar to that of SAG-treated wtMEFs or *Sufu*^-/- ^MEFs. Importantly, the wtMEFs and *Sufu*^-/- ^MEFs, which were ΔE1B variant-dominant, were both established from C57BL/6 mice, NIH3T3 cells, which were E1B variant-dominant were from BALB/c mice, while *Ptch1*^-/- ^MEFs, which had comparable expression of both variants, were from mice with mixed genetic background [22].

Overactivated HH signaling and Gli1 overexpression play a central role in medulloblastoma tumorigenesis [23]. This prompted us to investigate the expression profile of the Gli1 variants in medulloblastomas from the tumor-prone *Sufu*^+/-^*Trp53*^-/- ^mice maintained on a C57BL/6 genetic background (Heby-Henricson, K, Bergström, Å, Rozell, B, Toftgård, R, Teglund, S, unpublished) by using real-time RT-PCR (Figure 3C). As anticipated, the expression of all Gli1 variants was remarkably upregulated in the tumor samples compared to normal cerebellum, but the relative expression pattern of the variants was not apparently influenced by tumorigenesis. Additionally, the ΔE1B variant was found to be expressed at higher levels than E1B, as in wtMEFs and *Sufu*^-/- ^MEFs. Similar upregulation patterns of the Gli1 transcripts were also observed in medulloblastomas from *Ptch1*^+/- ^mice maintained on a C57BL/6 genetic background (See additional file 2).

These results are therefore suggesting that neither HH signaling activation nor tumorigenesis preferentially affect the regulation of expression of the individual Gli1 variants. On the other hand there is a strong influence of the mouse strain/genotype. C57BL/6 mice predominantly express the ΔE1B variant, BALB/c mice the E1B variant, while mice with mixed genetic background have comparable expression of both. In line with these observations, a mouse cDNA panel from BALB/c mice consistently showed increased expression of the E1B relative to the ΔE1B variant (data not shown).

### Identification of SNP and SINE polymorphisms involved in mouse Gli1 exon 1B inclusion/skipping

**Figure 4:**
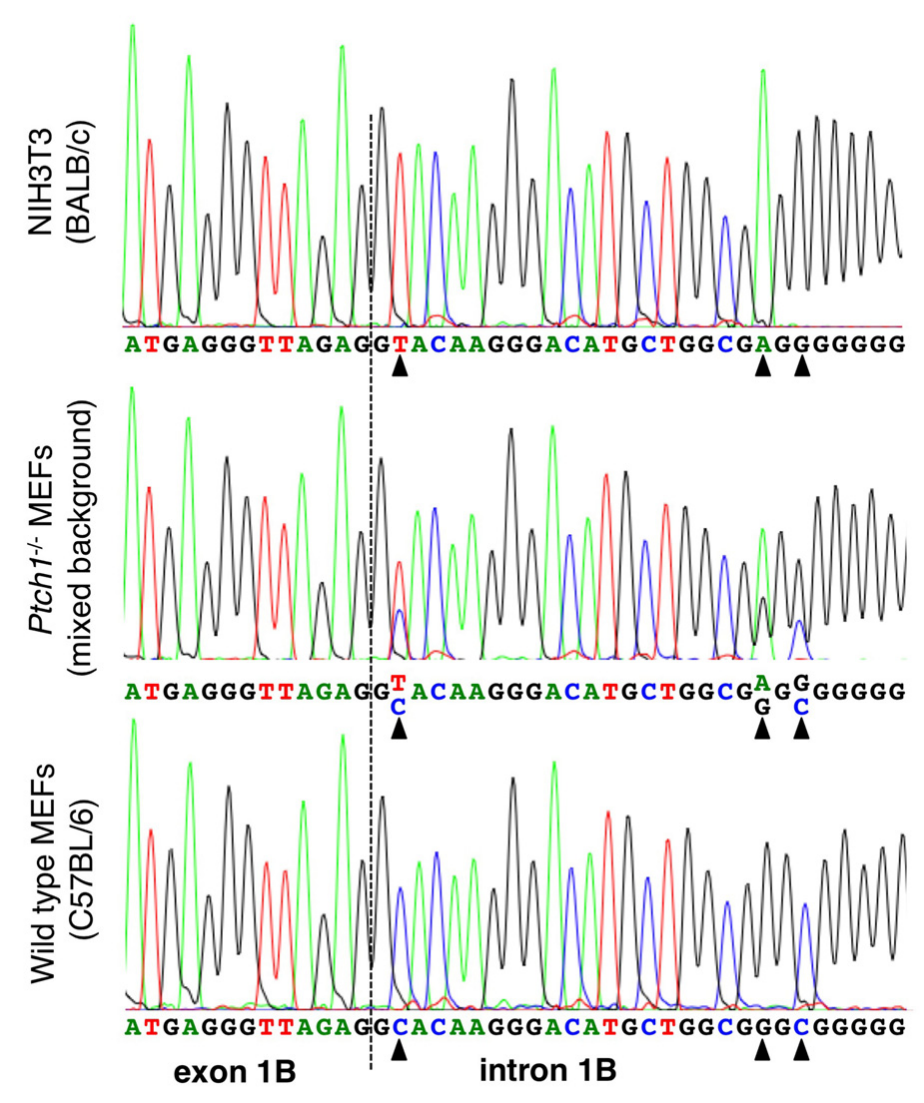
**Identification of genetic variations in the Gli1 exon 1B genomic region**. Electropherograms of the sequence analysis of the region spanning the 5' splice site of intron 1B. Genomic DNA from NIH3T3 cells, *Ptch1*^-/- ^MEFs and wtMEFs was PCR amplified and the products were analyzed by direct sequencing. The dotted line indicates the exon 1B/intron 1B boundary. The triangles highlight the SNP positions. Note the presence of the canonical GT intronic dinucleotide in NIH3T3 cells, the substitution to GC in wtMEFs, and the heterozygous GT/GC pattern in *Ptch1*^-/- ^MEFs.

Since our results clearly indicated that the relative expression pattern of the Gli1 variants was not affected by either HH signaling or tumorigenesis, but instead the ΔE1B to E1B ratio was well correlated with the mouse strains analyzed, variations in the genomic DNA sequence, such as single nucleotide polymorphisms (SNPs), could be involved. To identify differences among the mouse strains that influence the expression of these variants, we PCR amplified and sequenced the Gli1 exon 1B genomic region from NHI3T3, wtMEFs and *Ptch1*^-/- ^MEFs. Three SNPs in the vicinity of the donor site of the intron 1B were identified, one of which affected the conserved 5' intronic dinucleotides (Figure 4, DDBJ: AB520987 - AB520989). NIH3T3 cells, which predominantly express the exon 1B-included variants, have the canonical GT dinucleotide sequence at the intronic boundary. On the other hand, wtMEFs, as well as *Sufu*^-/- ^MEFs (data not shown), which predominantly express the exon 1B-skipped variants, have a GC intronic dinucleotide, implying a less efficient exon definition and consequently an increased exon skipping [24]. Interestingly, *Ptch1*^-/- ^MEFs are apparently heterozygous, containing both the GT and the GC alleles, in line with the comparable expression of the exon 1B-included and -skipped variants. Moreover, to further assess the effects of these SNPs on splicing efficiency, the GT and GC alleles were analyzed with two different splice site predictors, Human Splicing Finder [25] and NNSPLICE 0.9 [26]. Both programs demonstrated that the SNPs affect the prediction of splice sites, with the GT allele predicted to contain the expected 5' splice site, while this was not the case for the GC allele.

**Figure 5:**
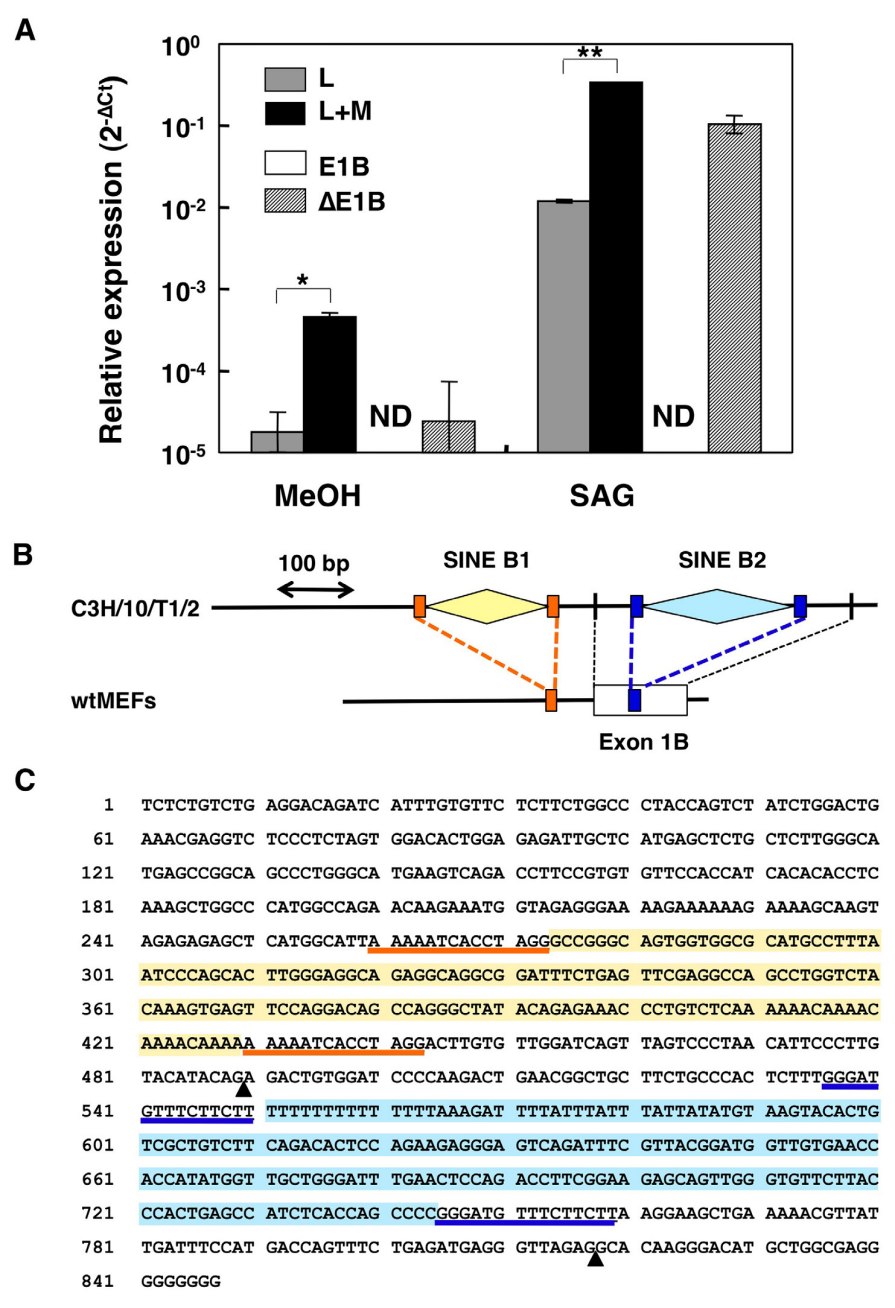
**The exon 1B structure/function in C3H/10T1/2 cells**. (A) Lack of the exon 1B-included Gli1 variants in the C3H/10T1/2 cell line. Gli1 variants in C3H/10T1/2 cells, treated with methanol (MeOH) or SAG, were analyzed by real-time RT-PCR. Data are presented as relative Ct (**C**ycle **t**hreshold) values (ΔCt), that is the Ct of individual transcripts minus the Ct of the housekeeping gene *Arp*. A logarithmic plot of the 2^-ΔCt ^values is shown. The PCR primer sets used are the ones depicted in Figure 2A. The error bars indicate the standard deviation and the statistical significance between the L and L+M as well as the E1B and ΔE1B transcripts is shown (*: p < 0.05, **: p < 0.01, Student's t-test). ND, non-detected, the signal is below the sensitivity limit of the assay. (B) Comparison of the genomic structure of the Gli1 exon 1B region in C3H/10T1/2 and wtMEF cells. The white box indicates the exon 1B, while the orange and blue boxes the "AAAAATCACCTAGG" and "GGGATGTTTCTTCTT" sequences, respectively, which are duplicated by the B1 SINE (orange rhomb) and B2 SINE (blue rhomb) insertions in C3H/10T1/2 cells. The vertical black bars in C3H/10T1/2 represent the intronic boundaries of exon 1B. The scale of the genomic sequence, 100 base pairs, is indicated by a double arrow. (C) Genomic sequence of the exon 1B region in C3H/10T1/2 cells. The SINE insertions and the duplicated regions are highlighted by the same colors as in (B). The triangles indicate the intronic boundaries of exon 1B.

Additionally, C3H/10T1/2 cells, which are generally used for HH signaling-dependent differentiation assays [27], were analyzed. Interestingly, no exon 1B inclusion could be detected by either real-time RT-PCR (Figure 5A) or nested PCR with primers within exon 1 and exon 4 (data not shown). Moreover, sequencing the exon 1B genomic region identified two insertions, a SINE B1 repeat in intron 1A and importantly, a SINE B2 repeat in exon 1B, which expanded the exonic sequence from 119 to 328 nucleotides (Figure 5B and 5C, DDBJ: AB520990). Consequently, the expanded exon 1B sequence, in combination with the presence of the GC intronic dinucleotide, further reduced exon definition, resulting in complete exon skipping [24]. These findings are therefore providing support to the notion of the importance of sequence polymorphisms in defining splicing patterns.

### Functional analysis of the Gli1 5' untranslated regions

**Figure 6:**
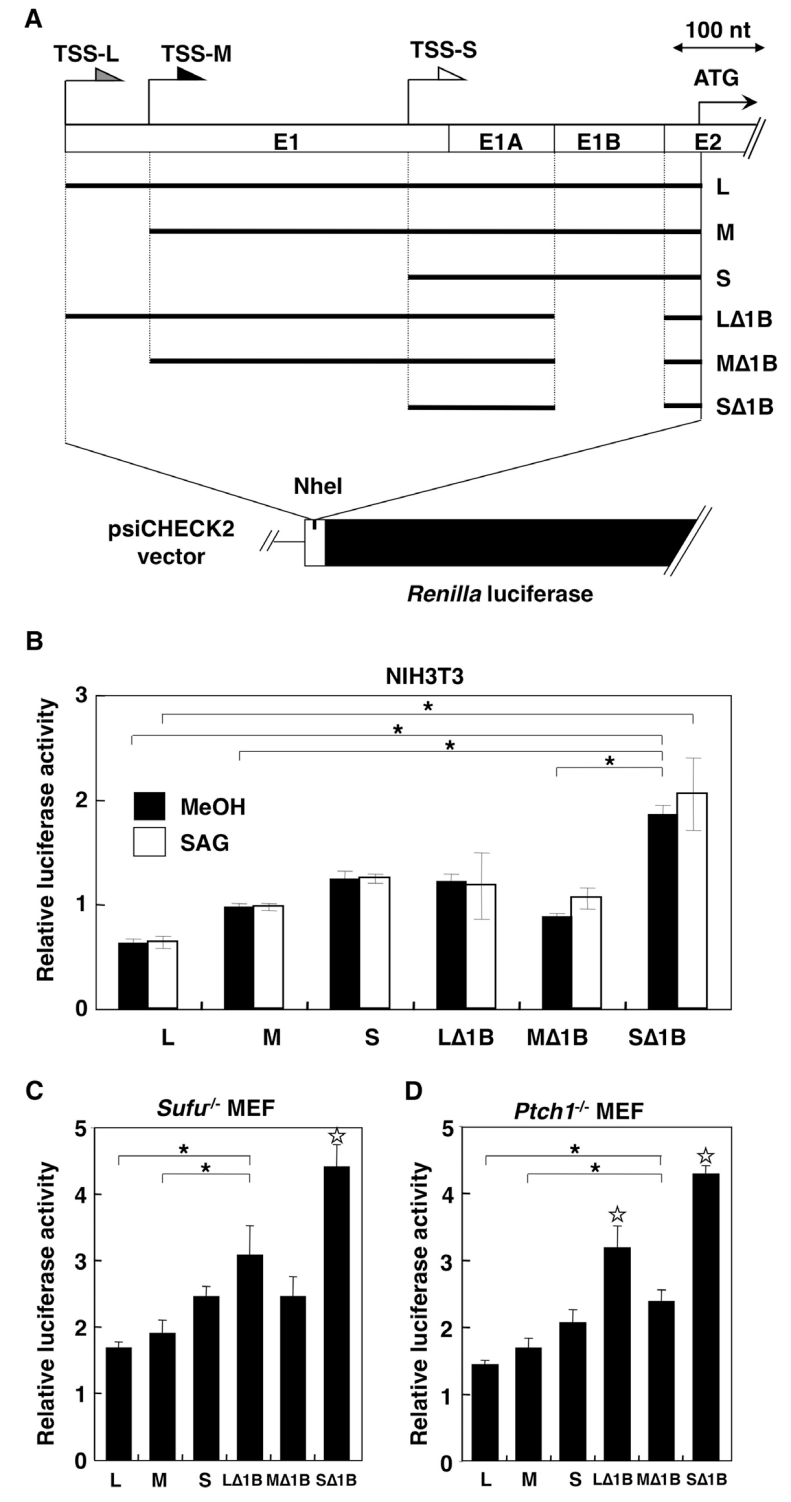
**Functional differences of the Gli1 5' UTR variants**. (A) Schematic representation of the six Gli1 5' UTRs analyzed. The alternative TSSs, the individual exons and the initiator methionine codon are represented as in Figure 2A. Bold lines indicate the six 5' UTR regions, L, M, S, LΔ1B, MΔ1B, SΔ1B, which were cloned into the NheI site, upstream of the *Renilla *luciferase coding region (black box) of the psiCHECK2 vector as described in methods. (B, C, and D) Luciferase activity of the six reporter constructs after transfection into NIH3T3 cells, *Sufu*^-/- ^MEFs, and *Ptch1*^-/- ^MEFs, respectively. Transfected NIH3T3 cells were treated with methanol (MeOH) or SAG. The *Renilla *luciferase activity was normalized relative to that of the *Firefly *luciferase. The error bars indicate the standard deviation. The statistical significance of the differences among the Gli1 5' UTR constructs is shown. (*: p < 0.01, ANOVA - Bonferroni test. The white star indicates significance relative to all other data sets).

Since the expression pattern of Gli1 variants was depended on the genetic background and was maintained as a fingerprint in different mouse strains, the possibility of regulatory roles for the alternative 5' UTRs was considered [6]. This was further supported by the fact that the 5' UTRs of mouse Gli1 have high GC content and are predicted to form multi- and long stem-loop structures (See additional file 3). To address the functional impact of these untranslated regions on the Gli1 mRNA transcripts, six 5' UTRs, namely L, M, S, LΔ1B, MΔ1B and SΔ1B (Figure 6A), were cloned in front of the *Renilla *luciferase gene of the psiCHECK2 vector. These constructs were transiently transfected into wtMEFs or NIH3T3 cells, with or without signaling activation by SAG treatment, and into *Ptch1*^-/- ^or *Sufu*^-/- ^MEFs. In all cases, elimination of 5' segments, from L to M and then to S resulted in higher luciferase activity (Figure 6B, C, D and See additional file 4). Additionally, skipping of exon 1B had a significant positive effect on the luciferase activity in the case of the L and S constructs, but was less pronounced for the M construct. This implies that not only 5' UTR length but also alterations in secondary structure, elicited by exon 1B skipping, may influence heterologous protein production. Moreover, HH signaling activation had no apparent influence on the observed luciferase activities. Collectively, these data suggest that the choice of transcription start site, in combination with exon 1B inclusion/skipping, influences the capacity of the Gli1 variants to produce a protein product.

Finally, we investigated whether this differential capacity of the 5' UTRs is due to modulations of the mRNA stability or the translational efficiency. To achieve this, the endogenous Gli1 levels in wtMEFs were enriched by SAG, followed by inhibition of the process of transcription with actinomycin D treatment and subsequent mRNA measurements. The results revealed no significant differences in mRNA stability among the Gli1 variants (See additional file 4). Thus, it may be suggested that the alternative 5' UTRs are likely to regulate Gli1 protein levels via translational mechanisms.

## Discussion

**Figure 7:**
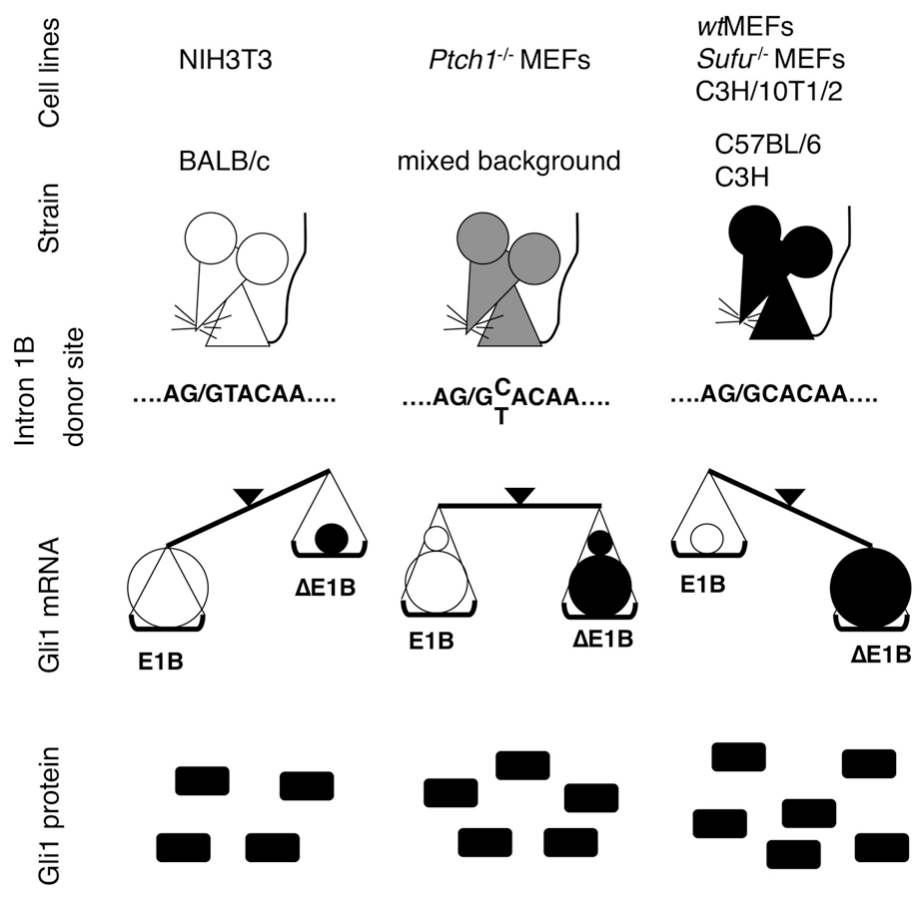
**Schematic model of the modulation of Gli1 protein levels by genetic variation**. The expression ratio of the Gli1 5' UTR variants is dependent on polymorphisms that affect the inclusion/skipping of exon 1B. The presence or not of the canonical intronic dinucleotide GT at the 5' splice site of intron 1B has a major impact in controlling exon 1B splicing. Additionally, the skipped variants have a higher translational efficiency, highlighting the importance of strain differences as modulators of HH signaling-dependent/independent Gli1 protein levels.

In this report the 5' UTR regions of the mouse *Gli1 *gene were analyzed, resulting in the identification of two novel transcription start sites and sequence polymorphisms, which control exon 1B inclusion/skipping. Our findings demonstrate that SNPs in the 5' splice site of intron 1B and a SINE B2 insertion in exon 1B have major effects in determining the expression ratio of the 5' UTR variant mRNAs, which distinctly influence translational efficiency (Figure 7).

Initially, 5' RACE and RT-PCR analysis clearly showed 5' variations in the Gli1 transcripts (Figure 1). However, the previously reported exon 2 - exon 3 skipping in human normal tissues/tumor cell lines [19], exon 3 - partial exon 4 skipping in human cancer cells and especially glioblastomas [20] and exon 1A - exon 1B skipping in mouse [18] could not be detected. Moreover, the 5' RACE analysis revealed the presence of some minor transcripts, apparently transcribed from alternative TSSs (data not shown, DDBJ: AB232673 - AB232676). As their expression levels were quite low, we chose to focus on the relatively abundant Gli1 variants in order to analyze the impact of alternative transcripts on HH signaling.

We demonstrated that exon 1B inclusion/skipping is intimately related with SNPs in the donor site of intron 1B (Figures 1, 2, 3, 4 and 5). The GT allele contains a canonical 5' splice site sequence, allowing efficient exon 1B definition and consequently enhances the inclusion of exon 1B, as observed in NIH3T3 cells. On the other hand, the GC allele has a weaker 5' splice site sequence, resulting in a less efficient exon 1B definition and therefore promotes the skipping of exon 1B, as observed in wtMEFs and *Sufu*^-/- ^MEFs. Moreover, *Ptch1*^-/- ^MEFs, which are heterozygous and contain both the GT and GC alleles, expressed comparable levels of the exon 1B-included and -skipped variants.

Since the relative expression pattern of the Gli1 variants was not affected by either HH signaling activity (Figure 3A and 3B) or tumorigenesis (Figure 3C)/embryogenesis (Figure 2B), the identified polymorphisms are primary determinants in controlling the splicing regulation of the Gli1 5' UTRs. SNPs/mutations affecting splicing have been reported for consensus splice sites, but were also identified at significant distances from splice junctions [28,29]. Our own analysis of the 284 reported mutations of *PTCH1 *revealed the presence of 20 intronic splice changes [30]. These findings highlight the importance of SNPs/mutations in altering splicing patterns, as these may occur not only at canonical splice sites but also at exonic/intronic splicing enhancers/silencers.

Additionally, evolutionary comparisons indicated that GC is likely to represent the ancestral intronic dinucleotide at the 5' splice site of intron 1B, since it is conserved in rat and other species, with the GT substitution and the SINE B2 insertion occurring at later stages (See additional file 5 and Figure 5). Interestingly, in the primate lineage the exon 1B region is characterized by an *Alu *insertion. These facts suggest that this genomic segment might be genetically unstable and a hotspot for transposon insertion.

5' UTRs are known to regulate protein expression via modulation of mRNA stability and/or translational efficiency [6]. We analyzed the alternative Gli1 5' UTRs in various cell lines and found that 5' end shortening as well as skipping of exon 1B increased their capacity for heterologous protein expression (Figure 6). These observations are in line with previous claims purporting that shorter 5' UTRs of Gli1 are more capable of efficient translation [18], and support the notion that alternative events in 5' UTRs of mammalian genes are likely to contribute to the regulation of translation [31]. Moreover, mRNA stability assays of the alternative Gli1 transcripts revealed that variations in the 5' UTRs did not affect the pattern of RNA degradation, and consequently, these untranslated sequences regulate Gli1 protein levels by modulating translational efficiency. Interestingly and in line with these observations, Hedgehog signaling-dependent mouse models for medulloblastoma development are apparently influenced by the Gli1 genotype. Deletion of one Ptch1 allele in C57BL/6 mice, which are homozygous for the GC allele, results in a higher incidence of medulloblastomas compared to mice with a mixed genetic background [32].

Secondary structure prediction of the Gli1 5' UTRs by Mfold highlighted the presence of a long stem-loop structure in L and M that is retained in MΔ1B but not in LΔ1B and may have a role in the differential translatabilities of MΔ1B versus LΔ1B (See additional file 3). However, free energy, ΔG, calculations were not fully in line with the capacity for translation. In addition, we examined whether two other parameters, G-quadruplex (G4) DNA/RNA structures and upstream ORF (upORFs), might influence the translational efficiency (See additional file 6). Nucleotide region 251-294 has a G4 motif sequence G_3_-N_1-7_-G_3_-N_1-7_-G_3_-N_1-7_-G_3_, and nucleotide region 22-55 has a similar sequence with a potential G4 structure. Moreover, the genome wide G4 DNA database QuadBase [33] predicted an antisense G4 motif at nucleotide region 322-360. G4-structures are formed not only on DNA but also on RNA [34], and a G4 motif on the NRAS mRNA was reported to suppress translation [35]. Additionally, the exon 1B-included variants, L, M and S, have an upORF in the same frame as the Gli1 ORF (fully upstream, 46 encoded amino acids), while the exon 1B-skipped variants, LΔ1B, MΔ1B and SΔ1B, have an upORF (38 encoded amino acids) that overlaps with the Gli1 ORF. Although no significant differences between fully upstream and overlapping upORF could be identified in a recent report, long cap-to-upORF distances were found to increase translational inhibition [36]. Wang and Rothnagel have used a 5' UTR construct (alfa-UTR), which is almost equivalent to the S construct in this report, mutated at four ATG codons and apparently eliminating 46 amino acids upORF that we have identified, and observed increased reporter activity, in line with the above predictions [18]. Thus, the combinations of upORFs with G4 structures in the Gli1 5' UTRs are likely to have a role as mediators of the observed patterns of translation.

## Conclusions

Our findings highlight the complex posttranscriptional regulation of the mouse Gli1 oncogene. mRNA variants with alternative 5' UTRs were identified, mechanisms that control their expression levels were dissected, and the differential impact of the 5' UTRs on protein synthesis was determined. Moreover, the demonstrated strain differences in regulatory controls of this oncogene suggest that these may have a role in modulating tumor susceptibility in mouse models.

## Methods

### RACE and PCR

5' RACE was performed by using the GeneRacer kit (Life Technologies, CA, USA), with mouse Gli1 exon 4 reverse primers (MWG-Biotech, Ebergsberg, Germany) (Table 1). The RACE products were analyzed on a 4% NuSieve 3:1 agarose gel (FMC BioProducts, ME, USA) and verified by PCR direct sequencing or sequencing of TA-clones in the pGEM-T vector (Promega, WI, USA). Pairs of initial and nested primers were also designed within mouse Gli1 exon 1, as shown in Table 1, and used in combination with the RACE exon 4 primers. The nested PCR analysis and sequence-verification were carried out as described in previous reports [37,38].

### Cell culture

The murine fibroblast cell lines NIH3T3, *Ptch1*^-/- ^MEFs [39], wtMEFs, *Sufu*^-/- ^MEFs and C3H/10T1/2 were cultured as described before [37,40,41]. Cells were treated with the **S**mo **ag**onist SAG at a concentration of 100 nM, with the medium changed to low serum (0.5% FBS or 1% FBS for wtMEFs), and allowed to grow for an additional 2 days.

### Mice

The use of animals was approved by the Stockholm South Animal Ethics Committee. The mice were kept at the animal facility of the Karolinska University Hospital, according to local and national regulations. The *Sufu*^+/-^*Trp53*^-/- ^mice were generated by intercrossing *Sufu*^+/- ^[41] and *Trp53*^+/- ^mice [42]. The *Ptch1*^+/- ^mouse strain has been described previously [22]. Both the *Sufu*^+/-^*Trp53*^-/- ^and the *Ptch1*^+/- ^strains were maintained on a C57BL/6 genetic background.

### Isolation of cerebellum cells

Normal cerebella and medulloblastoma tumors from *Sufu*^+/-^*Trp53*^-/- ^mice were digested with papain, triturated to obtain single-cell suspensions and then centrifuged through a 35%-65% Percoll gradient. Cells from the 35%-65% interface were suspended in Neurobasal medium (Life Technologies, CA, USA). Isolated granule cells were counted and checked with a microscope.

### RNA isolation, and real-time RT-PCR

Total RNA was isolated from cells, tissues and mouse embryos, using the RNeasy kit (Qiagen GmbH, Hilden, Germany) according to the manufacturer's protocol. Real-time RT-PCR was performed as described before [19]. Dissociation curves were generated after each PCR run to ensure that a single, specific product was amplified. The results were analyzed with the comparative **C**ycle **t**hreshold (Ct) method. For normalization, we used the expression level of Glyceraldehyde-3-phosphate dehydrogenase (Gapdh), and/or Acidic ribosomal protein (Arp). The PCR primers are shown in Table 2.

### Analysis of polymorphic variants

Genomic DNA was amplified with pairs of initial and nested primers, which are flanking the exon 1B region and are listed in Table 3. The PCR products were purified (Qiagen), and directly sequenced (MWG-Biotech). The obtained sequences were compared with the *Mus musculus *chromosome 10 genomic contig, strain C57BL/6J (GenBank: NT_039500).

**Table 3:** Primer sequences for polymorphism analysis

	Forward primers	Reverse primers
1st PCR	5'-TGGCGTGCCCTTCTGTTTCTTTGA	5'-TCCTGCAGGTTTCTGGGAGGTGTG
Nested PCR	5'-CGGGGAGACGCTCTGCTCTGAAGT	5'-TGGAGCCAGGTCTTTGAATGGGGAAT

### Functional analysis of 5' UTRs

For generating the 5' UTR constructs, we PCR amplified the selected regions by using specific RACE products as templates, with the primers (MWG-Biotech) listed in Table 4. The PCR products were then digested with the NheI restriction enzyme and cloned upstream of the *Renilla *luciferase open reading frame of the psiCHECK2 vector (Promega, GenBank: AY535007), which is under the control of the SV40 early enhancer/promoter. All constructs were verified by sequencing using BigDye Terminator v1.1 Cycle Sequencing Kits and an ABI prism DNA sequencer (Life Technologies).

**Table 4:** Primer sequences for generation of the 5' UTR constructs

	Forward primer	Reverse primer
L, LΔ1B	5'-GCG*GCTAGC*AGTAGGCAGTATAGGGTCC	5'-GCG*GCTAGC*GGCGTCTCAGGGAAGGAT
M, MΔ1B	5'-GCG*GCTAGC*TCCACCCAGCTCATCTTCTAG	5'-GCG*GCTAGC*GGCGTCTCAGGGAAGGAT
S, SΔ1B	5'-GCG*GCTAGC*CCAGCCCAGTTTCCAGCC	5'-GCG*GCTAGC*GGCGTCTCAGGGAAGGAT

The NheI restriction sites are shown in Italics.

Two hundred ng of each of the 5' UTR constructs were transfected into NIH3T3 or wtMEF cells, with or without SAG treatment initiated 24 hours after transfection, and *Ptch1*^-/- ^or *Sufu*^-/- ^MEFs using the FuGENE6 (Roche Diagnostics, Basel, Switzerland) transfection reagent. The activities of *Renilla *and *Firefly *luciferases were determined by using the dual-luciferase reporter assay system (Promega) with a FB12 Luminometer (Berthold Detection System, Pforzheim, Germany) or an Infinite M200 (Tecan, Männedorf, Switzerland) according to the manufacturer's recommendations. The experiments and individual measurements were performed at least twice.

## Abbreviations

Gli1: Glioma associated oncogene 1; HH: Hedgehog; Ptch: Patched; Smo: Smoothened; Sufu: Suppressor of Fused; UTRs: untranslated regions; RT: reverse transcriptase; MeOH: methanol; SAG: Smo agonist; Ct: cycle threshold; Gapdh: glyceraldehyde-3-phosphate dehydrogenase; Arp: acid ribosomal protein; d.p.c.: days post coitum; L: long 5' UTR transcript; M: medium 5' UTR transcript; S: short 5' UTR transcript; LΔ1B: L transcript with skipped exon 1B; MΔ1B: M transcript with skipped exon 1B; SΔ1B: S transcript with skipped exon 1B; SNP: single nucleotide polymorphism; G4: G-quadruplex.

## Authors' contributions

PZ and TS planned the work and supervised RP. RP was responsible for all experiments. ST and ML contributed to the experiments using mice. All authors read and approved the final version of the manuscript.

## Supplementary Material

Additional file 1 **Expression pattern of Gli1 variants in mouse embryos**. Additional figure 1, additional method, additional reference and additional table. Click here for file

Additional file 2 **Expression of Gli1 variants in medulloblastoma tumors**. Additional figure 2. Click here for file

Additional file 3 **Secondary structure characteristics of Gli1 5' UTRs**. Additional figure 3 and additional reference. Click here for file

Additional file 4 **Functional analysis of Gli1 5' UTRs**. Additional figure 4 and additional method. Click here for file

Additional file 5 **Comparison of the exon 1B/intron 1B junctions among different species**. Additional figure 5. Click here for file

Additional file 6 **G4 structures and upORFs in the Gli1 5' UTRs**. Additional figure 6. Click here for file
