# Supplementary material for: Genetic variations regulate alternative splicing in the 5' untranslated regions of the mouse glioma-associated oncogene 1, Gli1
Source: BMC Mol Biol. 2010 Apr 30;11:32. doi: 10.1186/1471-2199-11-32 (PMC2880320; doi:10.1186/1471-2199-11-32)
Supplement: Additional file 1 — Expression pattern of Gli1 variants in mouse embryos. Additional figure 1, additional method, additional reference and additional table. [file 1471-2199-11-32-S1.PDF]

Additional figure 1

A

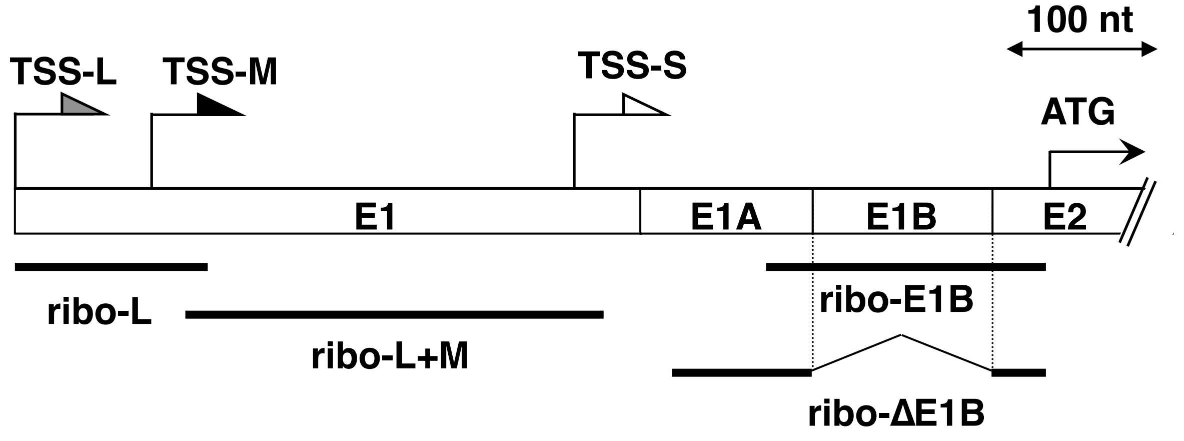

B

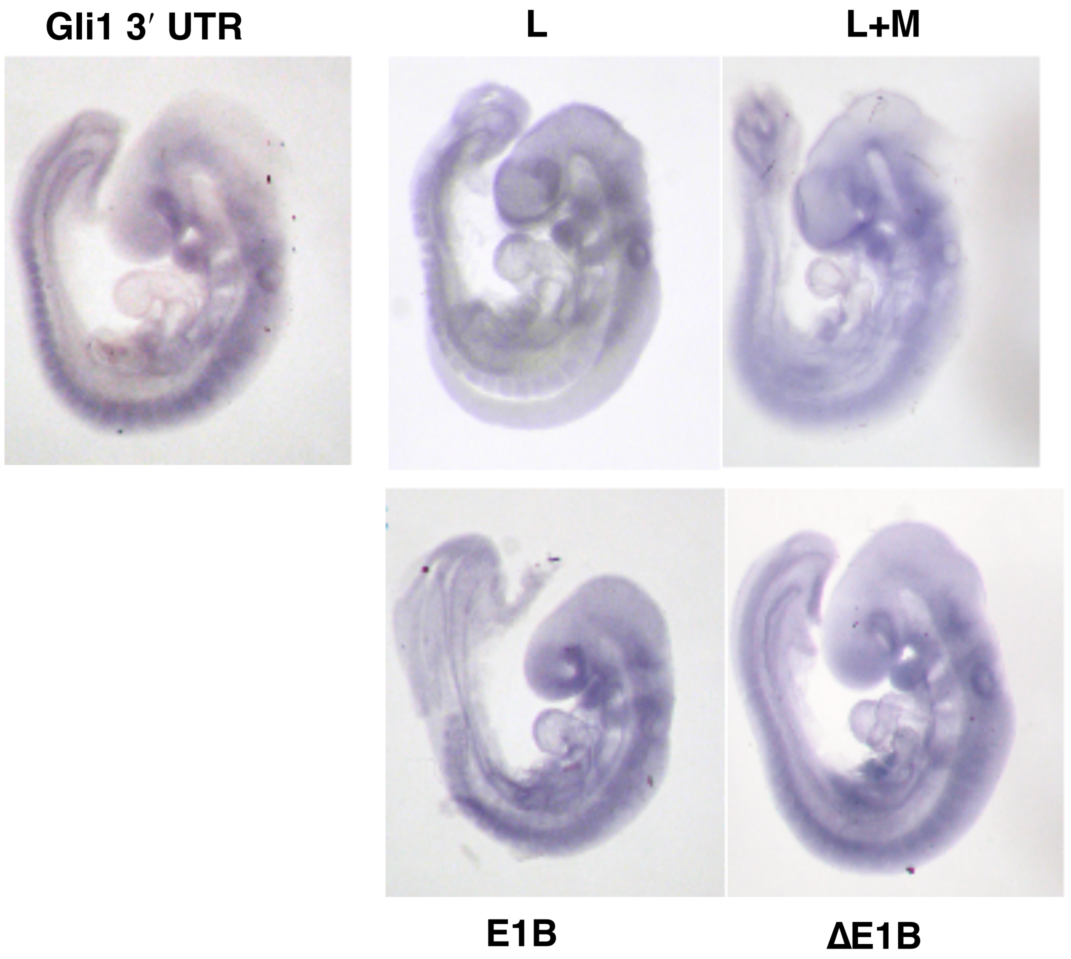

Additional figure 1. Expression pattern of Gli1 variants in mouse embryos. (A) Schematic representation of the variant-specific riboprobes, L, L+M, E1B, ΔE1B, (thick lines) used to detect the Gli1 transcripts. Gray, black and white arrows indicate the alternative transcription start sites TSS-L, TSS-M and TSS-S of the Gli1 mRNA. The initiator methionine codon (ATG) in exon 2 is also shown. (B) Distribution of Gli1 variants in embryos. Lateral view of whole mount *in situ* hybridization of 9.5 d.p.c. embryos with Digoxigenin (DIG)-labeled antisense probes. In addition to the L, L+M, E1B, and ΔE1B riboprobes, a Gli1 3′ UTR probe was also used as a control.

Additional method

Cloning of riboprobes and in situ hybridization

For construction of the Gli1 riboprobe plasmids, we amplified the selected regions by using the Advantage GC genomic PCR kit (Clontech, CA, USA) either on the mouse genomic BAC clone RP24-213L12 or on cDNA templates generated by 5′ RACE, with the primers listed in the Supplemental table. The PCR products were then cloned into the pGEM-T vector (Promega, WI, USA). All constructs were sequence-verified by using BigDye Terminator v1.1 Cycle Sequencing Kits and an ABI prism DNA sequencer (Life Technologies, CA, USA). As a control, a 3′ UTR probe corresponding to the Gli1 mRNA sequence (GenBank:AB025922) from nucleotide 1715 to 2266 was used [1].

9.5 d.p.c. C57BL/6 mouse embryos were fixed in 4% paraformaldehyde and hybridization was carried out according to standard procedures [1]. DIG-labelled probes (antisense and sense) were prepared using the DIG RNA labelling kit (Roche Diagnostics, Basel, Switzerland). The hybridized riboprobes were detected by the BM purple AP substrate (Roche Diagnostics). The specificity of the reaction was monitored by comparing the signals of the sense probes.

Additional reference

1. Svärd J, Heby-Henricson K, Persson-Lek M, Rozell B, Lauth M, Bergström A, Ericson J, Toftgård R, Teglund S: **Genetic elimination of Suppressor of fused reveals an essential repressor function in the mammalian Hedgehog signaling pathway.** *Dev Cell* 2006, **10(2):**187-197.

Additional table. Primer sequences for generating the riboprobes

Restriction sites are shown in Italics.

<table>
<tr><th></th><th>Forward primer</th><th>Reverse primer</th></tr>
<tr><td>L</td><td>5′-GCGCCATGGGTAGTAGGCAGTATAGGGTC</td><td>5′-GCGCTGCAGTTCCTTCGGACCGTTCTAGA</td></tr>
<tr><td>L+M</td><td>5′-GCGCCATGGGTCCGAAGGAAGGATATACG</td><td>5′-GCGGAGCTCTGGTCCAGGGCTGGAAACT</td></tr>
<tr><td>E1B</td><td>5′-TGGAGGTCTGCGTGGTAGA</td><td rowspan="2">5′-GATTGAACATGGCGTCTCAG</td></tr>
<tr><td>ΔE1B</td><td>5′-CCACATACTAGAAATCTCTCC</td></tr>
</table>
