# Supplementary material for: Genetic variations regulate alternative splicing in the 5' untranslated regions of the mouse glioma-associated oncogene 1, Gli1
Source: BMC Mol Biol. 2010 Apr 30;11:32. doi: 10.1186/1471-2199-11-32 (PMC2880320; doi:10.1186/1471-2199-11-32)
Supplement: Additional file 2 — Expression of Gli1 variants in medulloblastoma tumors. Additional figure 2. [file 1471-2199-11-32-S2.PDF]

Additional figure 2

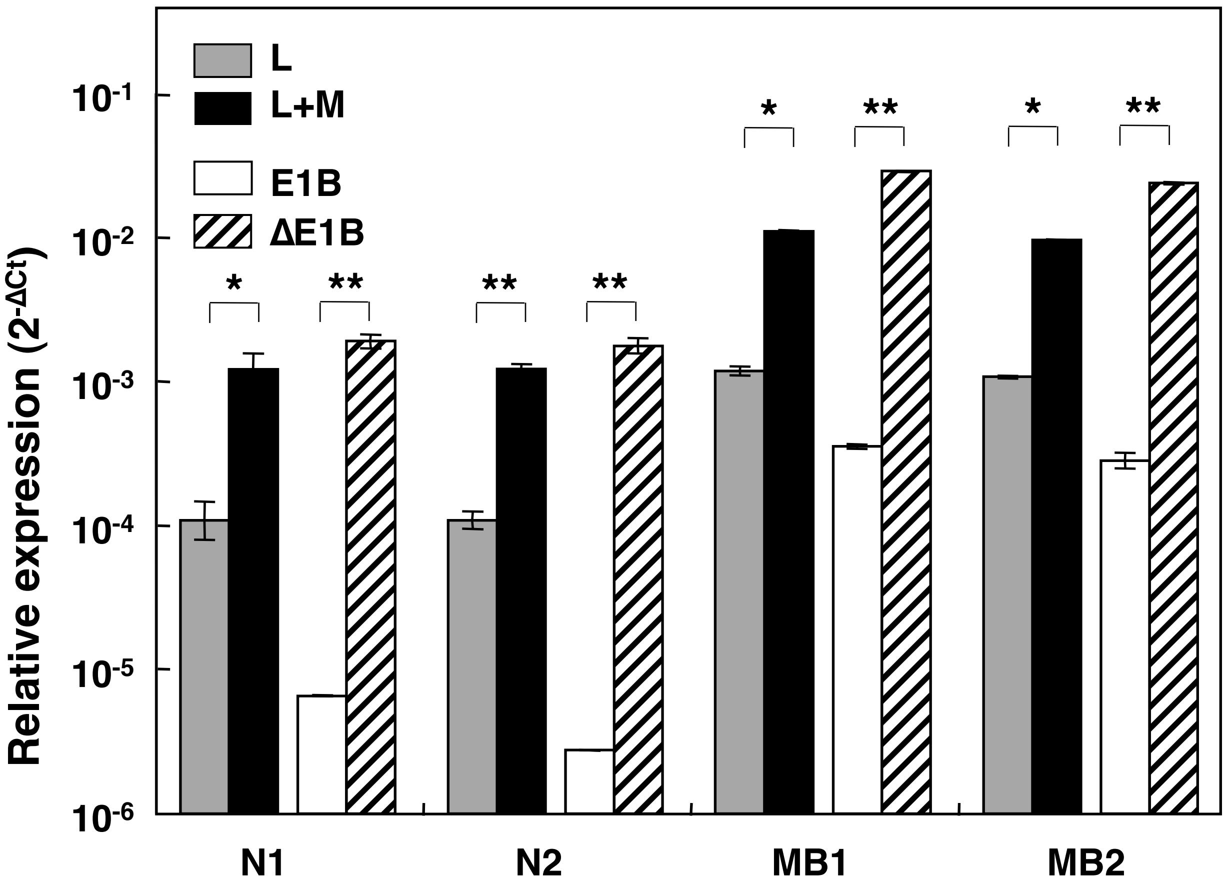

Additional figure 2. Expression of Gli1 variants in medulloblastoma tumors from *Ptch1*$^{+/-}$ mice. The expression profile in normal cerebellum (N1 and N2) and medulloblastoma tumors (MB1 and MB2) of *Ptch1*$^{+/-}$ mice was quantified by real-time RT-PCR using SYBR Green. Data are presented as relative Ct values (ΔCt), that is the Ct of individual transcripts minus the Ct of the housekeeping gene *Gapdh*. A logarithmic plot of the $2^{-\Delta Ct}$ values is shown. The PCR primer sets used are the ones depicted in Fig. 2A. The error bars indicate the standard deviation and the statistical significance between the L and L+M as well as the E1B and ΔE1B transcripts is shown (*: $p < 0.05$, **: $p < 0.01$, Student's t-test).
