# Supplementary material for: Genetic variations regulate alternative splicing in the 5' untranslated regions of the mouse glioma-associated oncogene 1, Gli1
Source: BMC Mol Biol. 2010 Apr 30;11:32. doi: 10.1186/1471-2199-11-32 (PMC2880320; doi:10.1186/1471-2199-11-32)
Supplement: Additional file 3 — Secondary structure characteristics of Gli1 5' UTRs. Additional figure 3 and additional reference. [file 1471-2199-11-32-S3.PDF]

Additional figure 3

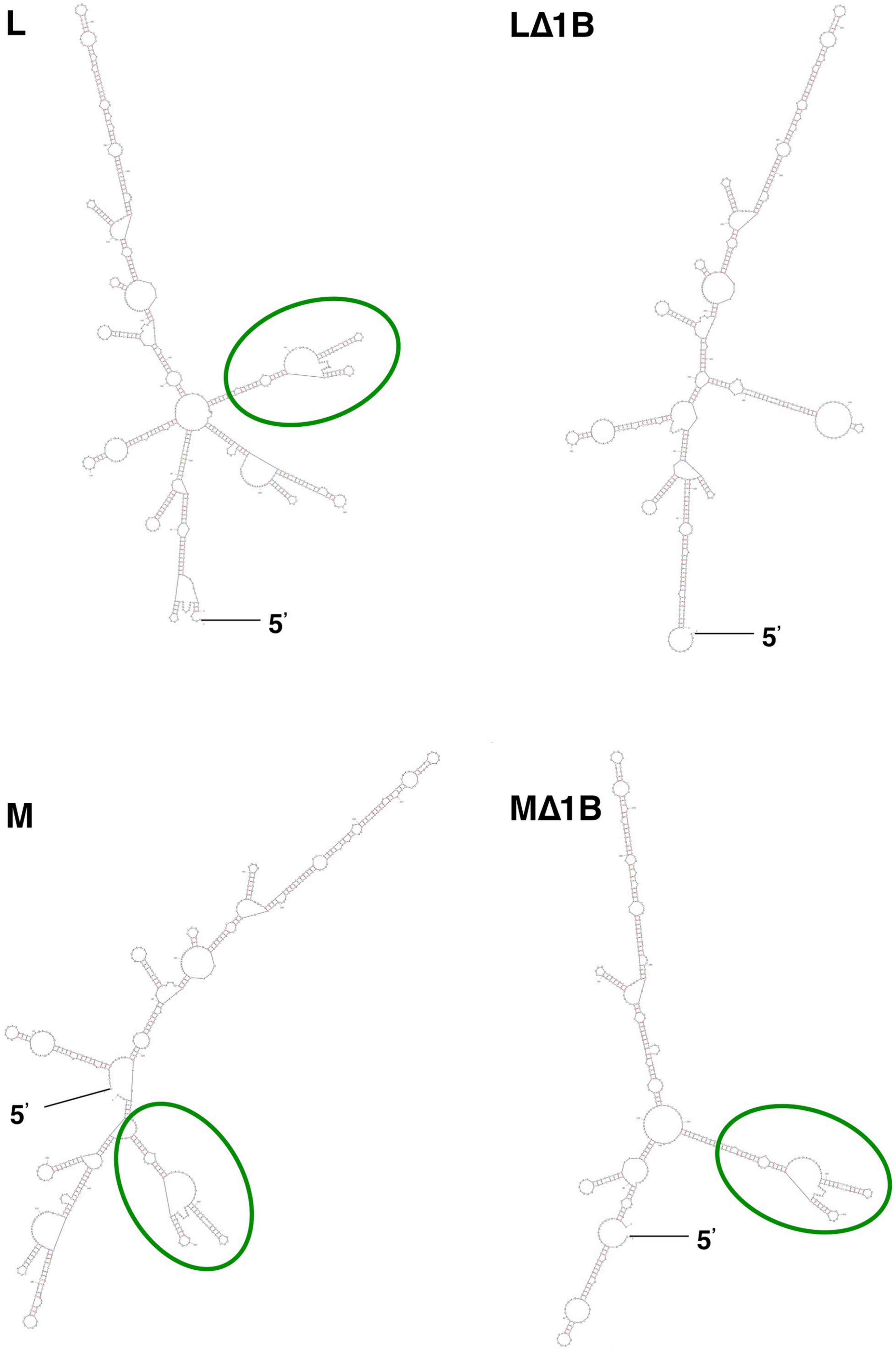

Additional figure 3

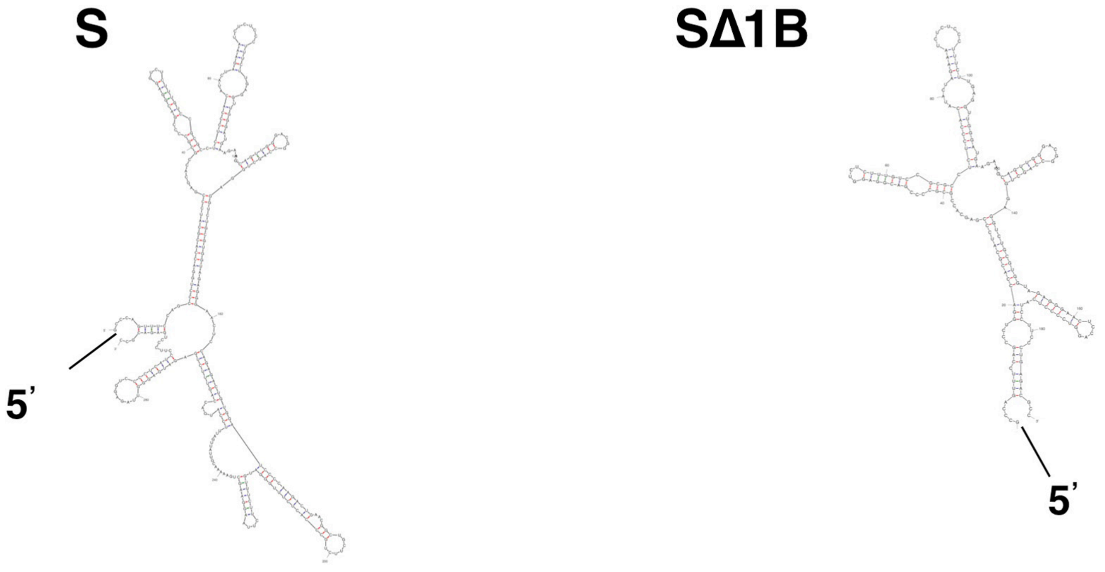

Additional figure 3. Secondary structure characteristics of Gli1 5′ UTRs. A visual representation of the secondary structures in the L, M and S 5′ UTR sequences, with or without exon 1B. The above structures were generated by the Mfold web-based server (http://mfold.bioinfo.rpi.edu/cgi-bin/rna-form1.cgi) [1]. Enclosed by a green line is the long stem-loop structure present in L, M and MΔ1B but not in LΔ1B.

Additional reference

1. Zuker M: **Mfold web server for nucleic acid folding and hybridization prediction.** *Nucleic Acids Res* 2003, **31(13):**3406-3415.
