# Supplementary material for: Genetic variations regulate alternative splicing in the 5' untranslated regions of the mouse glioma-associated oncogene 1, Gli1
Source: BMC Mol Biol. 2010 Apr 30;11:32. doi: 10.1186/1471-2199-11-32 (PMC2880320; doi:10.1186/1471-2199-11-32)
Supplement: Additional file 4 — Functional analysis of Gli1 5' UTRs. Additional figure 4 and additional method. [file 1471-2199-11-32-S4.PDF]

Additional figure 4

A

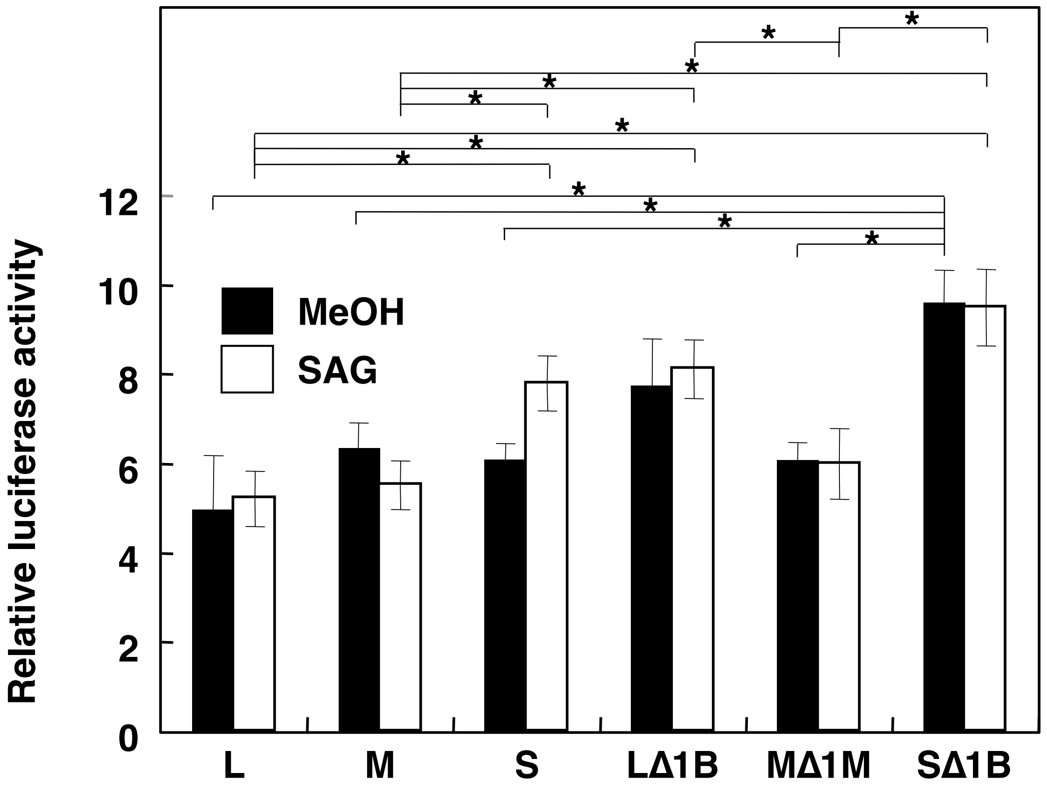

B

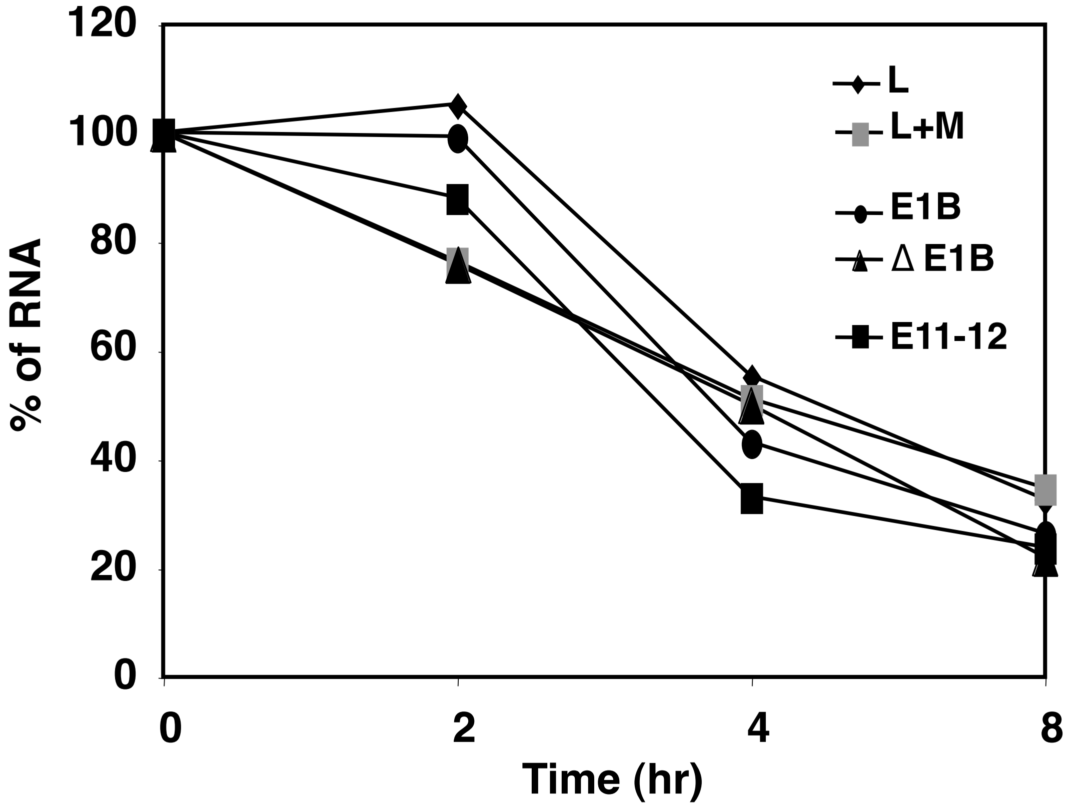

Additional figure 4. Functional analysis of Gli1 5′ UTRs. (A) Luciferase activity of the six 5′ UTR reporter constructs after transfection into wtMEFs and treatment with either methanol (MeOH) or SAG. The *Renilla* luciferase activity was normalized relative to that of the *Firefly* luciferase. The error bars indicate the standard deviation. The statistical significance of the differences among the Gli1 5′ UTR constructs is shown. (*: $p < 0.01$, ANOVA – Bonferroni test). (B) mRNA stability profile of Gli1 variants. WtMEFs were incubated with SAG and RNA was isolated following actinomycin D treatment for 0, 2, 4 and 8 hours. The expression of the Gli1 variants and the housekeeping gene *Arp* were detected by SYBR Green real-time PCR, with the primer sets of Table 2. For all Gli1 transcripts the mRNA levels at time point zero were set as 100%.

Additional method

Analysis of mRNA stability

WtMEFs were incubated with SAG for 48 hr as described in Materials and Methods. Then the cells were treated with the transcriptional inhibitor actinomycin D (Sigma Aldrich, MI, USA) at a concentration of 5 µg/ml and harvested at 0, 2, 4 and 8 hours after treatment. Total RNA was extracted and mRNA expression was quantified by real-time RT-PCR.
