# Supplementary material for: Genetic variations regulate alternative splicing in the 5' untranslated regions of the mouse glioma-associated oncogene 1, Gli1
Source: BMC Mol Biol. 2010 Apr 30;11:32. doi: 10.1186/1471-2199-11-32 (PMC2880320; doi:10.1186/1471-2199-11-32)
Supplement: Additional file 5 — Comparison of the exon 1B/intron 1B junctions among different species. Additional figure 5. [file 1471-2199-11-32-S5.PDF]

Additional figure 5

```
Mouse        TTTCTGAGATGAGGGTTAGAG/GCACAAGG
Rat          TCTCTGAGATGGGGGTCAGAG/GCACAAGG
Horse        GGTCTTGGA--GGGGCTGGGG/GCAC-ATG
Bushbaby     TATCTTAAA--GGGCCTGGAG/GCACAGGG
Tree shrew   TGTCTTAGA--GGAAATGGGG/GCATGGGT
```

Additional figure 5. Comparison of the exon 1B / intron 1B junctions among different species. All sequences presented are available in the NCBI database. The exon 1B / intron 1B boundary and the intronic dinucleotides are shown by a backslash and a red box, respectively. Note the presence of the rare intronic dinucleotide “GC” instead of the canonical “GT”.
