# Supplementary material for: Genetic variations regulate alternative splicing in the 5' untranslated regions of the mouse glioma-associated oncogene 1, Gli1
Source: BMC Mol Biol. 2010 Apr 30;11:32. doi: 10.1186/1471-2199-11-32 (PMC2880320; doi:10.1186/1471-2199-11-32)
Supplement: Additional file 6 — G4 structures and upORFs in the Gli1 5' UTRs. Additional figure 6. [file 1471-2199-11-32-S6.PDF]

Additional figure 6

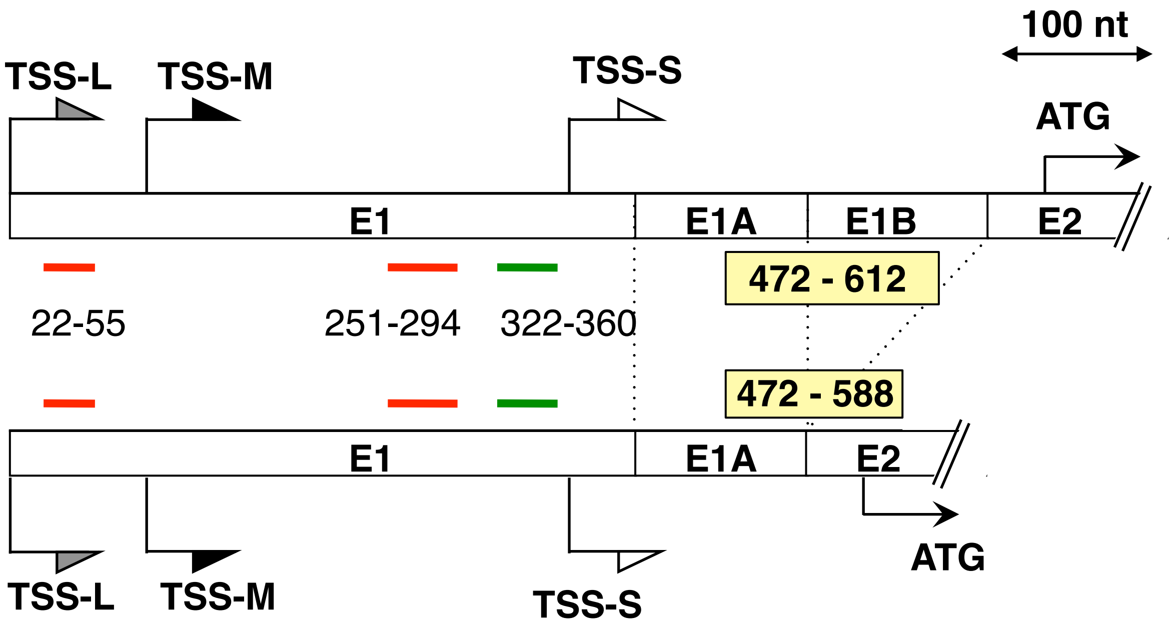

Additional figure 6. G4 structures and upORFs in the Gli1 5′ UTRs. Schematic representation of Gli1 5′ UTRs with or without exon 1B. G-quadruplex (G4) and antisense G4 motifs are indicated by red and green lines, respectively. UpORFs are shown in yellow boxes. Note that the upORF and the Gli1 ORF are in the same translational reading frame in the exon 1B-included variants, but in different frames in the exon 1B-skipped variants.
